# Post-Traumatic Stress, depression and anxiety symptoms among childhood cancer survivors and their parents

**DOI:** 10.1038/s41390-025-04724-4

**Published:** 2025-12-26

**Authors:** Maya Yardeni, Ilanit Hasson-Ohayon, Reut Pienik, Michal Mashiach-Eizenberg, Michal Ben Ami, Eve Stern, Hana Golan, Dalia Waldman, Amos Toren, Dalit Modan-Moses

**Affiliations:** 1 Division of Pediatric Hemato-Oncology, The Edmond and Lily Safra Children’s Hospital, Sheba Medical Center, Ramat-Gan, Israel; 2 Department of Psychology, Bar-Ilan University, Ramat-Gan, Israel; 3 Division of Pediatric Endocrinology and Diabetes, The Edmond and Lily Safra Children’s Hospital, Sheba Medical Center, Ramat-Gan, Israel; 4 Department of Health Systems Management, Yezreel Valley Academic College, Yezreel Valley, Israel; 5 Gray Faculty of Medical and Health Sciences, Tel Aviv University, Tel Aviv, Israel

## Abstract

**Background:**

Multiple studies demonstrated increased risk for psychological symptoms among childhood cancer survivors (CCS) and their parents. We aimed to assess the prevalence and severity of post-traumatic stress symptoms (PTSS), depression, and anxiety among CCS and their parents, examine associations between these measures, and identify potential risk factors.

**Methods:**

A cross-sectional study comprising 118 CCS aged 7–21 years and their parents, at least one year post-treatment. PTSS, depression and anxiety were assessed using validated questionnaires. Medical and sociodemographic data were collected from the medical charts.

**Results:**

PTSD criteria were met by 8.7% of CCS and 18.3% of parents, about a third reporting post-traumatic stress symptoms. Moderate-to-severe depression occurred in 22% of CCS and 7.6% of parents, and anxiety in 12.7% of CCS and 22% of parents. There were robust positive correlations between depression, anxiety, and PTSS within both CCS and parental self-reports. Parents’ proxy- reports correlated more strongly with their self-report than with CCS self-reports. Predictors of PTSS included time since diagnosis, parent’s education, parent’s sex, type of cancer, and depression and anxiety level.

**Conclusions:**

The study highlights the importance of screening both children and parents, prioritizing child self-report, and providing tailored, ongoing psychosocial support for CCS and their families.

**Impact:**

- Our study revealed high rates of psychological symptoms among CCS and their parents during the survivorship phase.
- The dual-informant design using pediatric self-report, parent self-report, and parent-proxy report enabled exploration of interconnections between psychological symptoms of CCS and their parents.
- Parents’ perceptions of children’s distress correlated more with their own emotional state than with children’s self-reports, suggesting parental projection.
- Predictors of post-traumatic stress symptoms, including time since diagnosis, parental education and biological-sex, depression and anxiety.
- Our results emphasize the need for screening both children and parents, prioritizing child self-report, and providing tailored, ongoing psychosocial support for CCS and their families.

## Introduction

The diagnosis of cancer represents a life-threatening experience, which is central to the concept of traumatic stress, according to the 5^th^ edition of the Diagnostic and Statistical Manual of Mental Disorders (DSM-5).^1^ This experience of traumatic stress can be due to an individual being diagnosed with cancer and undergoing treatment, or as a result of a loved one being diagnosed with the illness.

During the last five decades, there has been a dramatic improvement in survival rates of childhood cancer, and the five-year survival rates in developed countries increased from <20% to approximately 84% currently.^2,3^ However, it is estimated that 75% of childhood cancer survivors (CCS), develop complications related to their treatment years or even decades after completing therapy, which may evoke psychological difficulties such as anxiety, depression and trauma symptoms.^4,5^

Parallel to increased survival rates, numerous studies have shown that CCS and their parents are at risk to develop depression, anxiety and post-traumatic stress disorder (PTSD).^6^ Such emotional symptoms may be accompanied by neuroendocrine and immunological dysregulation. For example, studies assessing parents of pediatric cancer patients found that mothers with PTSS exhibited lower total urinary cortisol and a trend toward higher urinary norepinephrine levels,^7^ while parents with depression showed a lower CD4/CD8 T-cell ratio.^8^

There are many potentially traumatic events in the course of the acute treatment for cancer, including long hospitalizations, admissions to intensive care units, surgeries, intrusive procedures and sometimes, sadly, coping with the deaths of other children known to the family.^9^ All of these may evoke feelings of helplessness and intense fear among children with cancer and their families,^10^ and may result in PTSD, depression and anxiety.

### Anxiety and depression among CCS and their parents

When curative treatment has completed, CCS and their parents experience challenges with transitioning back to their previous life before the diagnosis. While many survivors and their families demonstrate resilience, a significant proportion experience clinically significant levels of anxiety and depression during and after treatment.^6,11–16^ Research indicates that approximately one-third of mothers of adolescent and young adult (AYA) cancer patients experience heightened psychological distress, such as depression and anxiety.^17^ Furthermore, it has been shown that 72% of mothers and 60% of fathers reported elevated psychological symptoms two months after their child completed treatment,^12^ while 20% of parents were diagnosed with anxiety and 14% with depression even five years post-treatment.^16^ Among CCS, 24% exhibited elevated anxiety and 29% experienced elevated depression three months after completing chemotherapy for ALL,^18^ a rate considerably higher than the general population. The remission stage, with its burden of physical, somatic, and psychological symptoms, is an especially vulnerable period.^15,19^ Factors contributing to this psychological burden include uncertainty about the future,^6,13,14^ fear of recurrence,^6,11,13,14^ treatment-related side effects, disrupted family routines, financial strain,^13^ severity of the child’s illness, intensity of treatment, negative affectivity and poor personal resources.^17^

### Post-Traumatic Stress symptoms (PTSS) among CCS and their parents

The growing body of literature from recent years consistently points to the significant presence of PTSS such as avoidance behaviors, intrusive thoughts, and hyperarousal, in both childhood cancer survivors (CCS) and their parents.^9,20^ However, determining the prevalence of PTSS is challenging due to the wide range of reported rates and methodological variations across studies. A review examining the prevalence of PTSS in CCS found that moderate to severe symptoms are present in 4.7–21% of survivors.^21^ Tremolada et al.^22^ found that 9.4% of adolescent and young adult CCS met the criteria for a full PTSD diagnosis, with an additional 11.2% presenting with subclinical levels of PTSS. In contrast, other investigators have described considerably lower PTSD rates among survivors, with no significant difference from controls.^23^

Reported rates of PTSS in parents of CCS are equally variable. However, determining the prevalence of PTSS is challenging due to the wide range of reported rates and methodological variations across studies. A review from 2011,^24^ for instance, found that PTSS prevalence rates among parents of CCS ranged from 12% to 80%, and PTSD rates from 6% to 34%. Similarly, van Warmerdam et al.^25^ in a meta-analysis of 58 studies, reported PTSD prevalence in parents of CCS between 4% and 75%. These inconsistent findings may be attributed to heterogeneity of study designs, assessment tools, and criteria for PTSD estimation, and further contribute to the challenge of establishing a clear understanding of the prevalence and impact of PTSS in CCS and their parents.^26^ A crucial insight emerged from a recent meta-analysis, which found that while depression and anxiety symptoms in CCS tended to decrease over time, PTSS remained relatively consistent after 12 months from cancer diagnosis.^27^ This underscores the potentially protracted nature of PTSS, highlighting the need for targeted interventions that address the long-term needs of CCS and their families.

### The relation between psychological symptoms of parents and their children

Several studies have revealed a significant correlation between the psychological well-being of CCS and their parents. De Castro et al.^28^ observed notable associations between specific PTSD subscales in CCS and their parents, particularly intrusion symptoms and negative alterations in mood and cognition. This suggests a potential shared experience of certain aspects of PTSS within families. Similar findings emerged from a study by Phipps et al.^29^, who reported significant positive correlations between children and parents’ self-reported PTSS. A longitudinal study by Sharp et al.^30^ further demonstrated similar, though weakly correlated, PTSS trajectories in children and their parents over a 5-year period, reinforcing the concept of interconnectedness in their psychological journeys. A meta-analysis by Bakula et al.^31^ provided compelling evidence for this connection, revealing strong correlations between a range of symptoms such as depression, anxiety, PTSS, and general distress in parents and children facing childhood cancer. However, despite the consistent finding of positive correlations across these studies, it is important to acknowledge certain methodological limitations that may have impacted the results. The range of time from diagnosis was highly variable, ranging from soon after diagnosis to several years later and some studies relied solely on parent reports of their child’s outcomes.^31,32^ These limitations underscore the need for further research that addresses these methodological issues to gain a more comprehensive understanding.

The aims of the current study were to evaluate the prevalence and severity of anxiety, depression, and PTSS in both CCS and their parents. In addition, to examine the associations between anxiety, depression, and PTSS in CCS and their parents and to identify risk factors for PTSS in CCS and their parents, including demographic characteristics (age, biological sex, socioeconomic status) and disease-related factors (cancer type, treatment intensity, time since diagnosis). Our study builds upon prior research through its design and scope. It employs a dual-informant design using pediatric self-report, parent self-report, and parent-proxy questionnaires; focuses on survivorship by including only CCS more than one year post-treatment rather than active cancer patients; examines dyadic correlations between parents’ and CCS reports; and explores predictors of PTSS in both CCS and their parents.

## Methods

### Study design

This study took place at the Pediatric Hemato-oncology Late Effects outpatient clinic, in a tertiary care center. It utilized a cross-sectional design using scales administrations for both children and parents.

### Participants and data collection

Participants and their parents were recruited during routine clinic visits. Inclusion criteria were child’s age 7 to 21 years at the time of reporting, at least 1 year from completion of medical treatment for any type of cancer at the time of enrollment. Exclusion criteria included inadequate comprehension of the Hebrew language by either the child or the parent, or an inability to independently complete self-assessment scales due to cognitive or other limitations. One-hundred and eighteen pairs of CCS and their parents were included in the current analysis, with a high response rate of 88%, and after removing a total of thirteen cases with significant missing data. The study was approved by the institutional ethical committee (5312-18-SMC) and informed consent was obtained from all parents as well as from CCS who were over 18 years of age. CCS and their parents were asked to complete measures separately and a research assistant was available to help with the completion of measures as needed. CCS completed self-report measures of depression, anxiety and PTSD, whereas parents completed both self-report and parent-proxy measures, as detailed below.

### Measures

*Depression & Anxiety* were assessed using the Patient Reported Outcomes Measurement Information System PROMIS)—An 8-item version of the depression and anxiety domains of the PROMIS Pediatric, Parent-proxy and Adult Short Forms.^33^ The PROMIS was developed by the NIH for use with both healthy populations and those with acute and chronic health conditions, with T-scores standardized to a mean of 50 and a standard deviation of 10. Ratings are made on a 5-point scale (0 = “not at all” to 4 = “very much” in the pediatric and parent proxy version; and 1 = “not at all” to 5 = “very much” in the adults’ version). The questionnaires have previously been translated into Hebrew and validated.^34^ In the present sample, internal consistency was high: for pediatric self-reports, Cronbach’s *α* = 0.937 (depression) and 0.871 (anxiety); for adult self-reports, *α* = 0.881 and 0.935; and for parent proxy-reports, *α* = 0.902 and 0.895 for depression and anxiety, respectively.

*Post-Traumatic Stress Symptoms* (PTSD and PTSS) *in CCS* were measured using the UCLA Children/Adolescents PTSD Reaction Index for *DSM-5* (PTSD-RI) self-report^35^ and caregiver report forms.^36^ This questionnaire has demonstrated strong evidence of validity, developmental appropriateness, and clinical utility in clinic-referred youth.^37^ The measure begins with an item which assesses lifetime exposure to traumatic events and includes a four-cluster criteria structure, following the DSM-5 symptom clusters, including intrusion (Cluster B), avoidance (Cluster C), negative alterations in cognitions and mood (Cluster D), and alterations in arousal and reactivity (Cluster E). The questionnaire consists of 27 items that are used to assess the four PTSD criteria (23 items) and the PTSD dissociative subtype (four items). Respondents rate symptoms on a 5-point Likert scale (ranging from 0 = “never happens” to 4 = “happens most of the time”). All items are summed to create a total severity score. A total score ≥ 33 was used as a cutoff indicating PTSD when describing prevalence of PTSD in CCS and in a regression analysis exploring predictors of trauma symptoms in CCS. Total severity score was used as a continuous variable for examining the correlation between PTSD, anxiety, and depression. The questionnaires were translated into Hebrew using a standard translation and back-translation procedure, and internal consistency in the present sample was high (Cronbach’s *α* = 0.915 for children/adolescents and 0.925 for caregivers).

*Post-Traumatic Stress Symptoms* (PTSD and PTSS) in parents were assessed using the Primary Care PTSD Screen for DSM-5 (PC-PTSD-5), a five-item screen that was designed to identify individuals with probable PTSD in primary care settings. The measure begins with an item which assesses lifetime exposure to traumatic events. If a respondent denies exposure, the PC-PTSD-5 is complete with a score of 0. However, if respondents indicate that they have had any lifetime exposure to trauma, they are instructed to respond to five additional yes/no questions about how that trauma exposure has affected them over the past month.^38^ The standard cutoff score of ≥ 3, was employed to classify individuals as screen-positive for PTSD. Reporting two symptoms was considered indicative of PTSS. The questionnaire has been previously translated to Hebrew and validated^39^ and internal consistency in the present sample was acceptable (Cronbach’s α = 0.766).

#### Medical and sociodemographic characteristics

Parents completed a demographic questionnaire assessing background characteristics including age, sex and ethnicity of their child, and marital status and educational level of parents. Information about the child’s diagnosis, date of diagnosis, details of the medical treatment and date of end of cancer treatment was obtained from the medical chart.

#### ITR -3 scale

The Intensity of Treatment Rating Scale 3.0 (ITR-3) is a well-established empirical approach for grading the intensity of pediatric cancer treatments. It classifies 34 types of cancer and treatments modules (e.g., chemotherapy, radiation, surgical operation, stem cell transplantation) into the four levels from 1 - least intensive treatment to 4 - most intensive treatment.^40,41^ The level of treatment was determined according to patients’ medical chart.

### Data analysis

Analyses were conducted using the IBM SPSS Statistics 28.0. Missing values were less than 0.5% and were imputed by replacing them with the mean of the available data within the scale. Given the negligible extent of missing values, a formal sensitivity analysis was not performed, as such a small percentage of missing values is unlikely to bias statistical results. This approach is consistent with methodological guidance suggesting that missing data rates below approximately 5% are considered trivial.^42–45^ Cronbach’s α coefficient was measured to verify the reliability of the measurement tools used in the study. Analyses were performed in three steps. First, descriptive analyses were conducted to describe the participants’ demographic and clinical characteristics. Second, Pearson correlations were performed to explore relationships between the study variables. Finally, we conducted a hierarchical multiple regression analysis to test the contribution of all variables to the prediction of PTSD in CCS and the parents. Significance was set at the 0.05 level.

## Results

### Participants’ characteristics

Demographic and clinical characteristics of the study participants are presented in Table 1. The majority of participating parents were mothers (72.9%), born in Israel (84.7%), with an academic degree (54.2%). Fifty-four percent of participating CCS were females. Mean age at the time of diagnosis was 7.49 ± 4.85 years (range 0–18 years) and mean age at the time of reporting was 14.72 ± 3.79 years (range 8.1–22 years). The most common diagnoses were leukemia (33.1%), and brain tumors (33.1%). The majority of patients (62.7%) were diagnosed more than 5 years prior to the study (mean time from diagnosis 7.24 ± 4.11 years).

**Table 1:** Demographic and clinical characteristics of the study sample (*n* = 118).

		*N*	%
CCS Sex	Female	64	54.2%
	Male	54	45.8%
CCS age at diagnosis (range)	0–18 years	118	
CCS age at reporting (range)	8.1–22 years	118	
Reporting parent	Mothers	86	72.9%
	Fathers	32	27.1%
Mother’s country of origin	Israel	100	84.7%
	Other	18	15.3%
Father’s country of origin	Israel	103	87.3%
	Other	15	12.7%
Mother’s Education^a^	>12 years	2	1.7%
	12 years	22	18.6%
	Academic degree	64	54.2%
	Other degree	20	16.9%
Father’s Education^b^	>12 years	5	4.2%
	12 years	39	33.1%
	Academic degree	44	37.2%
	Other degree	18	15.3%
Religious Affiliation^c^	Secular	53	44.9%
	Traditional	21	17.8%
	Religious	13	11.0%
	Orthodox	21	17.8%
Diagnosis	Leukemia	39	33.1%
	Lymphoma	18	15.3%
	Brain tumor	39	33.1%
	Other solid tumor	22	18.6%
ITR levels	ITR 1	2	1.7%
	ITR 2	43	36.4%
	ITR 3	42	35.6%
	ITR 4	31	26.3%
Time from diagnosis	Up to 5 years	44	37.3%
	More than 5 years	74	62.7%

*CCS* Childhood Cancer Survivors, *ITR* Intensity of treatment rating.

^a^Missing: 10 (8.5%), ^b^Missing: 12 (10.2%), ^c^Missing: 10 (8.5%).

### Trauma, depression and anxiety of CCS and their parents

**Fig. 1:**
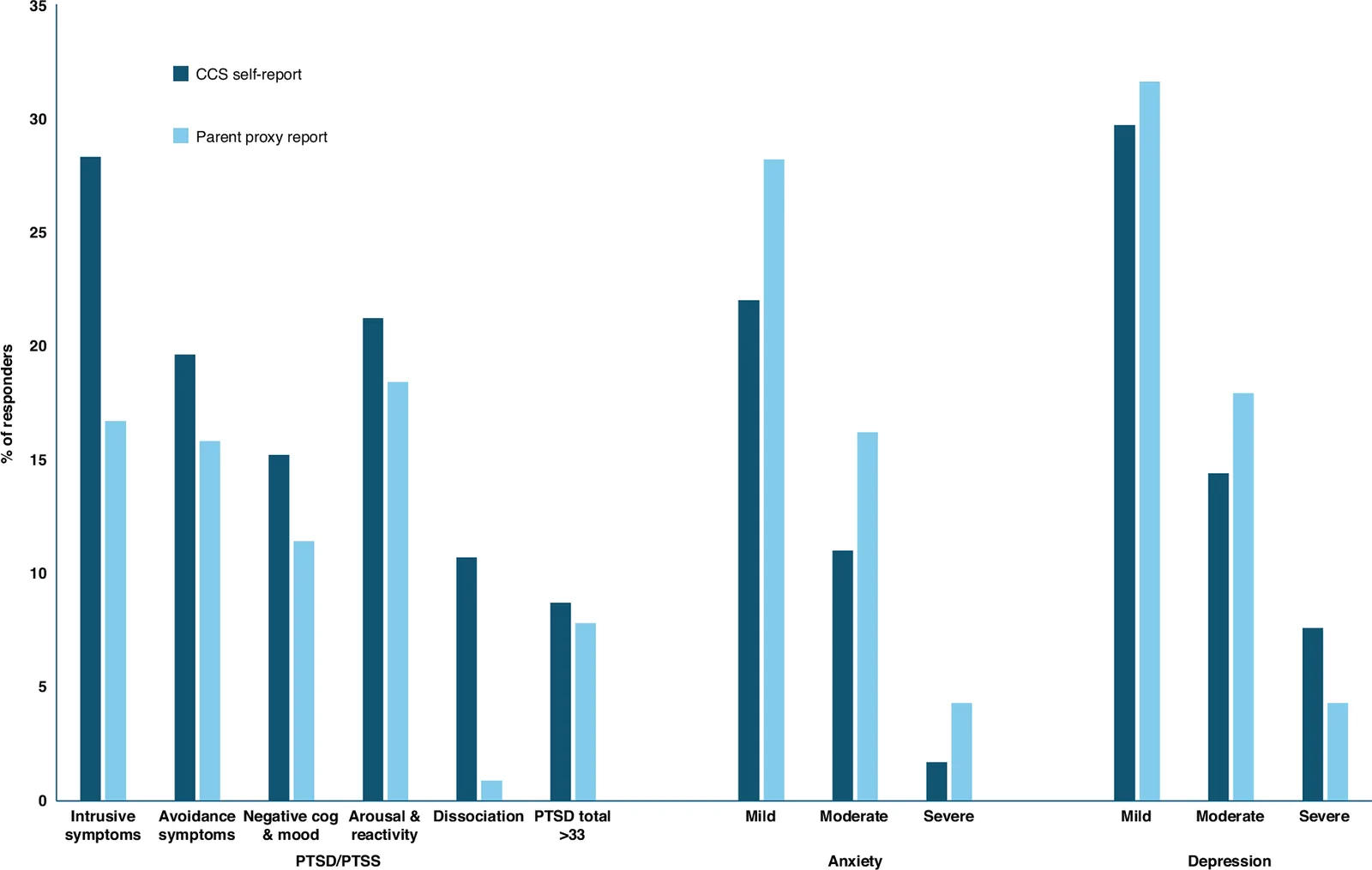
Prevalence of PTSS, anxiety, and depression: CCS self-reports vs parent-proxy reports. Clustered bar chart showing the prevalence of PTSS, anxiety, and depression based on child self-reports and parent-proxy reports. Parents reported lower levels of trauma-related symptoms and higher levels of anxiety and depression in their children compared to children’s self-report.

Rates of clinically significant symptoms of trauma, depression and anxiety according to the CCS self-report, parents’ proxy report and parents’ self-report questionnaires, are presented in Table 2. By the UCLA-PTSD self-report questionnaire, 8.7% of CCS met the criteria for a full diagnosis of PTSD. Examining the distinct symptom domains, almost a third of our patients (28.3%) reported suffering from intrusive symptoms, 21.2% reported arousal and reactivity symptoms, and 19.6% reported avoidance symptoms. Observing the parents’ PC-PTSD questionnaires, 18.3% of them were screen-positive for PTSD while almost a third (32.1%), reported suffering from two symptoms of post trauma. Table 2 also depicts the depression and anxiety rates of participants according to the PROMIS questionnaires. Twenty-two percent (22%) of CCS had PROMIS self-report scores consistent with moderate to severe levels of depression, similar to the parents’ proxy reports (22.2%). Rates of moderate to severe levels of anxiety were 12.7% by the CCS self-reports, whereas by the parents’ proxy report they were 20.5%. Observing parents’ self-reports, 7.6% reported moderate to severe levels of depression, and 22% moderate to severe levels of anxiety. As illustrated in Fig. 1, parents generally reported lower levels of trauma-related symptoms than children, yet higher rates of anxiety and depression, highlighting informant discrepancies.

**Table 2:** Frequencies of depression, anxiety and trauma symptoms (*n* = 118).

	Pediatric self-report	Parent proxy- report	Parent self-report
PTSD :			
B -Intrusive symptoms (≥1)	28.3%	16.7%	
C - Avoidance symptoms (≥1)	19.6%	15.8%	
D - Negative cog +Mood (≥2)	15.2%	11.4%	
E - Arousal + Reactivity (≥2)	21.2%	18.4%	
A - Dissociation (≥1)	10.7%	0.9%	
PTSD Total ≥ 33	8.7%	7.8%	
PTSD – PC PTSD - 5 ( ≥ 2)^a^			32.1%
(≥3)^b^			18.3%
Anxiety^c^ (mild > 55)	22.0%	28.2%	35.6%
(moderate > 60)	11.0%	16.2%	14.4%
(severe > 65)	1.7%	4.3%	7.6%
Depression^c^ (mild > 55)	29.7%	31.6%	21.2%
(moderate > 60)	14.4%	17.9%	6.8%
(severe > 65)	7.6%	4.3%	0.8%

*PTSD* post-traumatic stress disorder, *UCLA* The University of California at Los Angeles Posttraumatic Stress Disorder Reaction Index, *PROMIS*, Patient reported outcomes measurement information system, *PC-PTSD 5* Primary Care PTSD.

^a^Reporting two symptoms was considered indicative of PTSS.

^b^Reporting three symptoms or more was considered screen-positive for PTSD.

^c^According to PROMIS T-Scores.

The correlations between parents’ self-reports, parents’ proxy-reports, and pediatric self-reports scores of depression, anxiety, and PTSD, are presented in Table 3. Parent self-reports of depression, anxiety, and PTSD were strongly correlated with each other. Similarly, pediatric self-reports of depression, anxiety, and PTSD were inter-correlated, as were the parent-proxy scores for the three conditions. In addition, there were positive and significant correlations between parents’ self-reports and parents’ proxy-reports for all three conditions. The correlations between parent proxy-reports and pediatric self-reports, although significant, were considerably weaker, suggesting a gap between children’s self-reports and the parents’ perception of their child’s PTSS, depression and anxiety symptoms. Finally, the correlations between parent self-reports and pediatric self-reports were weak, and were mostly significant for the PTSD scores.

**Table 3:** Pearson correlations between study measures (*N* = 118).

Parent self-report				Parent proxy-report			Pediatric self-report	
	Depression	Anxiety	PTSD	Depression	Anxiety	PTSD	Depression	Anxiety
Parent self–report Anxiety	0.82***							
Parent self–report PTSD	0.55***	0.57***						
Parent-proxy Depression	0.49***	0.45***	0.29**					
Parent-proxy Anxiety	0.55***	0.53***	0.36***	0.66***				
Parent proxy PTSD	0.52***	0.45***	0.50***	0.65***	0.74***			
Pediatric self-report Depression	0.14	0.19*	0.21*	0.33**	0.23**	0.26**		
Pediatric self-report Anxiety	0.01	0.12	0.16	0.15*	0.22**	0.20*	0.65***	
Pediatric self-report PTSD	0.22**	0.23**	0.25**	0.37***	0.24**	0.38***	0.75***	0.60***

*p < .05, **p < .01, ***p < .001.

### Multiple regression analysis predicting trauma symptoms in CCS and parents

Two hierarchical multiple linear regressions were used to predict trauma symptoms (PTSD scores) in CCS and their parents. As can be seen in Table 4, in step 1, the following socio-demographic variables were entered into the regression: sex, time from cancer diagnosis, fathers’ and mothers’ education, and type of cancer (leukemia, lymphoma and brain tumor, referred to solid tumors). In step 2, variables of PROMIS anxiety and depression parents’ self-report, parent-proxy reports and pediatric self-reports, were entered.

**Table 4:** Hierarchical multiple regressions for predicted CCS and parent PTSD.

	UCLA - PTSD Pediatric (*N* = 105)				PC-PTSD - PTSD Parent (*N* = 98)			
Predictor Variable	*B*	*SE*	*β*	*R*^2^	*B*	*SE*	*β*	R^2^
Step 1:								
Constant	33.39	4.55		0.24	0.86	0.60		0.25
Sex - child/parent *(female* = *1)*	2.77	2.32	0.11		1.31	0.32	0.39***	
Time from diagnosis	−0.91	0.30	−0.31**		−0.04	0.04	−0.11	
Religious *(traditional&orthodox* = *1)*	−3.00	2.36	−0.12		0.22	0.30	0.07	
Leukemia^a^	−7.03	3.22	−0.27*		−0.42	0.40	−0.13	
Lymphoma^a^	−8.88	4.26	−0.25*		−0.90	0.52	−0.21	
Brain tumor^a^	−5.15	3.31	−0.20		−0.88	0.41	−0.27*	
ITR 3^b^	−4.41	2.91	−0.17		0.10	0.36	0.03	
ITR 4^c^	−3.55	3.17	−0.13		0.06	0.40	0.02	
Father’s education	1.42	2.79	0.06		0.91	0.37	0.30*	
Mother’s education	−6.68	3.28	−0.23*		−0.71	0.42	−0.20	
Step 2:								
Constant	14.10	3.97		0.64	−1.08	0.66		0.43
Sex - child/parent *(female* = *1)*	−0.47	1.73	−0.02		0.95	0.29	0.28**	
Time from diagnosis	−0.41	0.22	−0.14		−0.02	0.03	−0.05	
Religious *(traditional&orthodox* = *1)*	−0.82	1.68	−0.03		0.13	0.26	0.04	
Leukemia^a^	0.13	2.38	0.01		−0.35	0.35	−0.11	
Lymphoma^a^	−1.96	3.11	−0.05		−0.56	0.47	−0.13	
Brain tumor^a^	−2.03	2.50	−0.08		−0.61	0.37	−0.19	
ITR 3^b^	−0.96	2.08	−0.04		0.13	0.32	0.04	
ITR 4^c^	−0.37	2.31	−0.01		−0.04	0.35	−0.01	
Father education	−0.75	2.00	−0.03		0.59	0.33	0.20	
Mother education	−4.22	2.41	−0.14		−0.31	0.38	−0.09	
PROMIS Parent proxy - anx	−0.27	0.21	−0.12					
PROMIS Parent proxy - dep	0.65	0.27	0.24*					
PROMIS Pediatric - anx	0.46	0.19	0.21*					
PROMIS Pediatric - dep	0.85	0.16	0.49***					
PROMIS Adult - anx					0.07	0.04	0.30*	
PROMIS Adult - dep					0.06	0.05	0.19	

*B* Unstandard coefficient, *SE* Standard Error, *β* Standard coefficient.

*p < .05, **p < .01, ***p < .001.

^a^The reference group is Solid tumors.

^b^The reference group is ITR 1 + 2.

^c^The reference group is ITR 1+2+3.

#### Predictors of PTSS in CCS

In step 1, time since diagnosis was a significant negative predictor, meaning that CCS further out from diagnosis reported lower PTSD symptoms. Having a diagnosis of either leukemia or lymphoma, was also a significant negative predictor, suggesting that children with these diagnoses reported lower levels of PTSD. Mother’s education was another negative predictor for children’s PTSD in the first step. Biological sex, religious affiliation and the intensity of medical treatment (ITR) score did not appear to play a significant role in predicting PTSD in CCS. In step 2, the significant predictors for PTSS were parents’ proxy reports for depression and the children’s self-reports for anxiety and depression. In step 1, the predictors explained 24% of the variance of PTSD score in CCS. The addition of depression and anxiety measures in step 2, raised the explained variance for CCS PTSD score from 24% to 64%.

#### Predictors of PTSS in parents

In step 1, being female sex was a significant positive predictor for trauma symptoms (i.e., mothers reported higher levels of PTSD symptoms). Father’s education was also a positive predictor of parent-reported PTSD in the first step. A diagnosis of Brain tumor diagnosis was a significant negative predictors for parents’ PTSD. Religious affiliation, time from diagnosis and ITR score were not found to be significant predictors for PTSD among parents. In step 2, after adding PROMIS scores, anxiety self-report score was identified as a significant predictor for parents’ PTSD score. In step 1, the predictors explained 25% of the variance in parents’ PTSD score, and the addition of depression and anxiety measures increased the explained variance from 25% to 43% of parents PTSD score.

## Discussion

The primary purpose of the current study was to promote a better understanding of the psychological challenges of CCS and their parents during the survivorship phase.

Our study revealed considerable rates of psychological symptoms among CCS and their parents during the survivorship phase. The finding that 8.7% of CCS met full PTSD diagnostic criteria aligns with a previous study by Tremolada et al.^22^, who reported similar prevalence rates. The rates of PTSD symptoms are particularly noteworthy, with 28.3% of CCS reporting intrusive symptoms, 21.2% experiencing arousal and reactivity symptoms, and 19.6% demonstrating avoidance symptoms. These rates highlight the profound psychological impact of childhood cancer that extends well beyond the active treatment period. Similarly, 18.3% of parents were screen-positive for PTSD and 32.1% had PTSS, underscoring the significant psychological burden experienced by parents. As noted by Phipps et al.^23^, when strict diagnostic methods are applied, PTSD prevalence among children with cancer may resemble that of community samples. However, subclinical PTSS remain common and clinically important, reflecting ongoing distress and functional difficulties even in the absence of a full diagnosis. These findings are consistent with previous literature indicating that the diagnosis and treatment of childhood cancer are profoundly traumatic and intrusive experiences, not only for the children but also for their parents.^24,25,46–48^ The depression and anxiety rates were equally concerning. Twenty-two percent of CCS reported moderate to severe depression, with similar rates observed in parent-proxy reports, however, parents’ self-report rate for moderate to severe depression was lower (7.6%). In contrast, anxiety rates among parents were higher, with 22% of parents self-reporting moderate to severe anxiety, compared to 12.7% in CCS self-reports. The difference in rates of depression and anxiety between parents and children at the survivorship phase is likely due to the different and unique challenges they experience. Based on previous studies, exploring the experience of loneliness among CCS,^14,32^ and studies investigating parents coping with the fear of cancer recurrence,^6,13,49^ understanding the unique struggles of both children and parents and the discrepancies between them during this period, emphasizes the need to provide support tailored to their specific needs.^50,51^

A significant contribution of our study is the exploration of interconnections between trauma, depression, and anxiety symptoms. Within both CCS and parental self-reports, we observed robust positive correlations between depression, anxiety, and PTSD scores. This finding is consistent with existing literature on the comorbidity of psychological symptoms.^28,31^ The pattern of correlations suggests that parents’ perceptions of their children’s emotional state are more closely related to their own emotional state than to their children’s self-reported experiences. This discrepancy can be understood in terms of parental projection, where parents’ own emotional state influences interpretation of their children’s emotional expressions. The lower correlations between parent proxy-reports and pediatric self-reports underscore the importance of obtaining information directly from children, since relying solely on parental reports, as done in some prior studies,^31,32^ may not accurately capture the child’s emotional experience.

Our regression analyses identified predictors of trauma symptoms in both CCS and their parents, highlighting the complex interplay of factors associated with depression and anxiety symptoms following cancer diagnosis. Regarding socio-demographic and disease-related variables, we found that mothers’ education and a diagnosis of leukemia or lymphoma were associated with lower levels of PTSD in CCS. This finding contrasts with previous research that did not identify specific cancer types as a predictive risk factor.^47,50^ Unlike Kazak et al.,^9^ in our analysis the severity of the diagnosis and of the treatment were not identified as direct predictors of post-traumatic stress. Our analysis also revealed that longer time since the cancer diagnosis was associated with lower reports of PTSS among CCS, as previously observed by other investigators.^47^ This finding might be explained by the concept of posttraumatic growth (PTG). PTG is a frequently reported positive outcome of childhood cancer, when over time, survivors may find meaning in their experience, develop a greater appreciation for life, and experience increased self-worth, which may lead to decreased psychological symptoms.^27,50,52^

Among parents, female sex was a significant positive predictor of trauma symptoms, which aligns with findings from Sultan et al.^17^ Father’s education was also a positive predictor of trauma symptoms in parents, which contradicts some prior studies.^52^ In addition, and also surprisingly, a diagnosis of a brain tumor was a negative predictor of trauma symptoms for parents, a finding that is inconsistent with the literature.^9,17^ An explanation for these results may lie in our reliance on CCS self-reports, which limited us to assessing children whose brain tumor did not limit their cognitive abilities. Consistent with previous studies,^50,52^ we found that anxiety and depression were significant positive predictors for PTSD in CCS. Similarly, and in line with the findings from previous studies,^50^ the analysis indicates that parents’ self-reported anxiety was a significant positive predictor of parents’ trauma symptoms.

These results highlight the necessity to consider the unique factors that influence psychological symptoms in both children and their parents, and to develop targeted interventions for families facing childhood cancer.

### Strength and limitations

Our study provides a comprehensive evaluation of trauma, depression, and anxiety symptoms in CCS and their parents, offering a holistic view of their emotional vulnerability during the survivorship phase. The study also explores the interconnections between psychological symptoms of CCS and their parents, as well as parents’ perceptions of their child’s emotional state, highlighting the complex family dynamics at play. The use of validated questionnaires, and the high response rate of 88% increase the reliability and validity of our findings. Additionally, our findings have clinical relevance and can be applied to the hospital settings, where CCS and their parents come for follow-up care. However, the study also has limitations. The sample was drawn from a single center, and included only Hebrew-speaking participants, factors that may limit the generalizability of our findings to other settings and cultural contexts. Nevertheless, our tertiary center treats patients from all over the country. Furthermore, there was a disproportionate representation of mothers, probably due to the fact that mothers are more likely to accompany their child to the hospital. The cross-sectional design limits the ability to determine causality between the factors. The use of self-report and proxy-report measures may be subject to informant bias, as the indicated levels of depression, anxiety and trauma symptoms may not always match the evaluations of mental health professionals. Finally, the UCLA PTSD-RI was translated to Hebrew for use in the current study and has not been previously validated in this language. Future studies will benefit from applying longitudinal design and integrating objective assessment of psychopathology.

### Conclusions and implications

The current study underscores the long-term psychological impact of childhood cancer on both survivors and their parents, highlighting the need for comprehensive mental health interventions. The high rates of trauma, anxiety, and depression found in both CCS and their parents call for early and continuous mental health support to be integrated into survivorship care plans. Our findings also suggest the need for routine screening for psychological symptoms in both CCS and their parents. Specifically, we suggest that child self-report should be prioritized as the primary indicator of CCS psychological symptoms, while parent-proxy reports should be interpreted cautiously and in the context of the parent’s own distress levels. Furthermore, interventions should be tailored to the unique needs of both children and parents as they face different challenges during the survivorship phase. For example, CCS are more vulnerable to depression, struggling with the transitioning from the inclusive environment during their medical treatment into expectation and life demands and might require support with transitioning back into their normal life and social groups. However, parents cope with different challenges, such as worrying about cancer recurrence, their child’s future health, and the impact of the cancer journey on the family as a whole including financial issues, and as a result have higher rates of anxiety. By addressing these challenges with interventions like psychological counseling, support groups and stress management programs, the well-being and quality of life for CCS and their families can be improved.

## Data Availability

The data that support the findings of this study are available upon request from the senior author. The data are not publicly available because of privacy or ethical restrictions.
